# A mathematical model to predict “low-lying” posterior communicating artery aneurysms in neurosurgical practice

**DOI:** 10.1007/s10143-024-02638-z

**Published:** 2024-08-01

**Authors:** Qianquan Ma, Lei Liu, Zhihao Song, Hongbo Wen, Kaihuan Li, Jingqi Chen, Weixin Zhang, Tao Huang, Yufeng Xu, Haoyu Li, Ting Lei, Xuezhi Sun

**Affiliations:** 1 Department of Neurosurgery, Peking University Third Hospital, Peking University, Beijing, China; 2 School of Mathematics and Statistics, Central South University, Changsha, Hunan China; 3 Department of Neurosurgery, Xiangya Hospital, Central South University, Changsha, Hunan China; 4 Department of Neurosurgery, Yiyang Central Hospital, Yiyang, Hunan China; 5 Department of Neurosurgery, Sanbo Brain Hospital, Capital medical university, Beijing, China

**Keywords:** PcoA, “low-lying” PCoA aneurysms, Predict model, Anterior clinoid process, Anterior petroclinoid Fold

## Abstract

“Low-lying” posterior communicating artery (PCoA) aneurysms require great attention in surgical clipping due to their distinct anatomical characteristics. In this study, we propose an easy method to immediately recognize “low-lying” PCoA aneurysms in neurosurgical practice. A total of 89 cases with “low-lying” PCoA aneurysms were retrospectively analyzed. All patients underwent preoperative digital subtraction angiography (DSA) examinations and microsurgical clipping. Cases were classified into the “low-lying” and regular groups based on intraoperative findings. The distance- and angle-relevant parameters that reflected the relative location of the aneurysms and tortuosity of the internal carotid artery were measured using 3D-DSA images. The data were sequentially integrated into a mathematical analysis to obtain the prediction model. Finally, we proposed a novel mathematical formula to preoperatively predict the existence of “low-lying” PCoA aneurysms with great accuracy. Neurosurgeons might benefit from this model, which enables them to directly identify “low-lying” PCoA aneurysms and make appropriate surgical decisions accordingly.

## Introduction

Intracranial aneurysms are more likely to arise from the bifurcation or curvature of the cerebral vessels. The posterior communicating artery (PCoA) is the most common site of internal carotid artery (ICA) aneurysms, which account for 20–30% of all intracranial aneurysms [1]. Although endovascular treatments have been the first choice for treating cerebral aneurysms, some patients receive microsurgical clipping, especially for very small aneurysms, giant aneurysms, and aneurysms with complex aneurysmal neck shapes [2]. Successful microsurgical clipping requires sufficient space for proximal control and complete exposure of the aneurysmal neck. Compared with aneurysms at other sites, some “low-lying” PCoA aneurysms require particular attention in clinical practice [3].

“Low-lying” PCoA aneurysms were first addressed by Kim in 2009 [4]. These peculiar aneurysms present at fairly short distances from the anterior clinoid process (ACP) and anterior petroclinoid fold (APF). Overhanging ACPs are usually large enough to block the aneurysmal necks and the proximal portion of the ICAs in “low-lying” PCoA aneurysms. The occluded aneurysmal neck and proximal ICA caused by the ACP and APF cause more difficulties and risks in clipping procedures. Additional APF fenestration or anterior clinoidectomy is required to release the operative space and gain access to the proximal ICA and aneurysmal neck [5, 6]. In this regard, accurately identifying “low-lying” PCoA aneurysms prior to surgery is essential to assess the surgical risks. This study proposes an evaluation model to preoperatively predict “low-lying” PCoA aneurysms based on digital subtraction angiography (DSA) data. This novel system will enhance the understanding of anatomic characteristics of “low-lying” PCoA aneurysms and provide valuable guidance for clinical practice.

## Patients and methods

### Patients

This retrospective study enrolled patients with ruptured PCoA aneurysms who were treated between January 2020 and December 2023 at our center. All patients underwent preoperative DSA to confirm the presence of aneurysms. A total of 89 patients who underwent microsurgical clipping were included in the analysis. All the patients consented to the procedure. All cases were subdivided to the “low-lying” or regular groups based on intraoperative findings.

### Radiographical parameters

All parameters in this prediction system were obtained from two aspects. Distance-relevant parameters represented the relative locations of the aneurysms and origin of the PCoA. Angle-relevant parameters represented the tortuosity of the ICA.

### Distance-relevant parameters

**Fig. 1:**
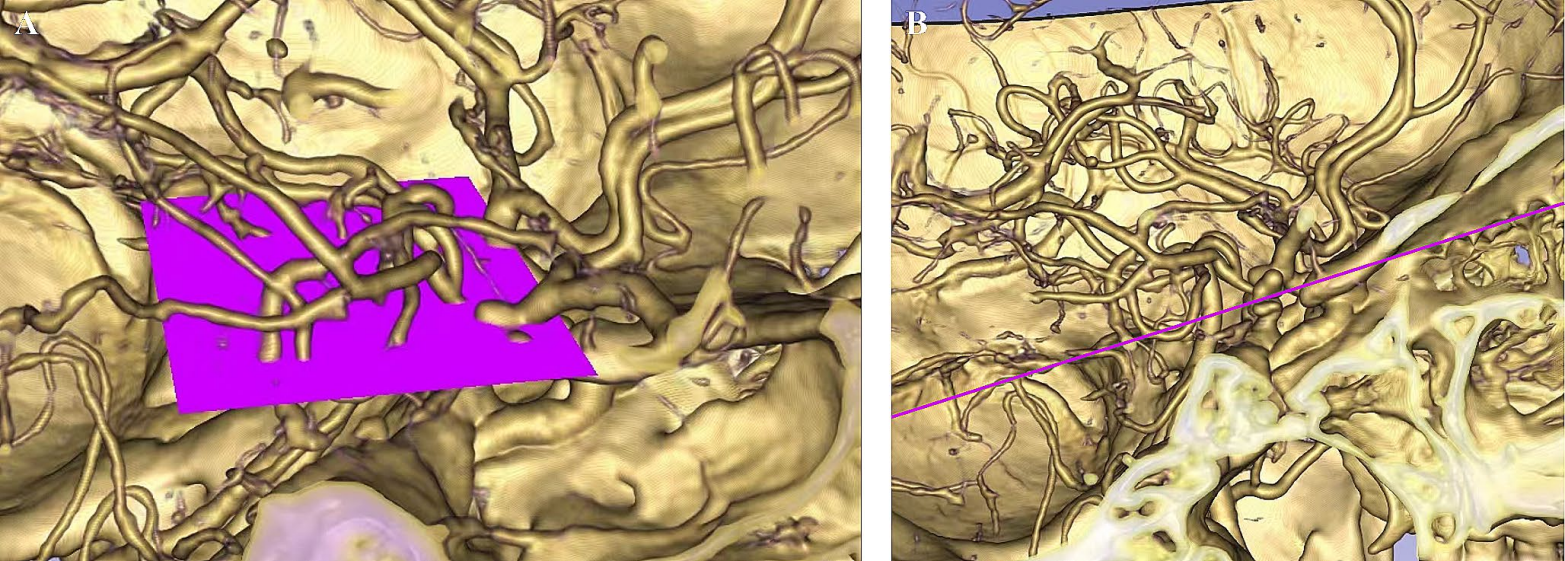
Diagram of SF. (**A**) The SF is an imaginary surface which crosses the bilateral ACP tips and is parallel to the planum sphenoidale. (**B**) Sagittal view, the pink line represents the level of SF

**Fig. 2:**
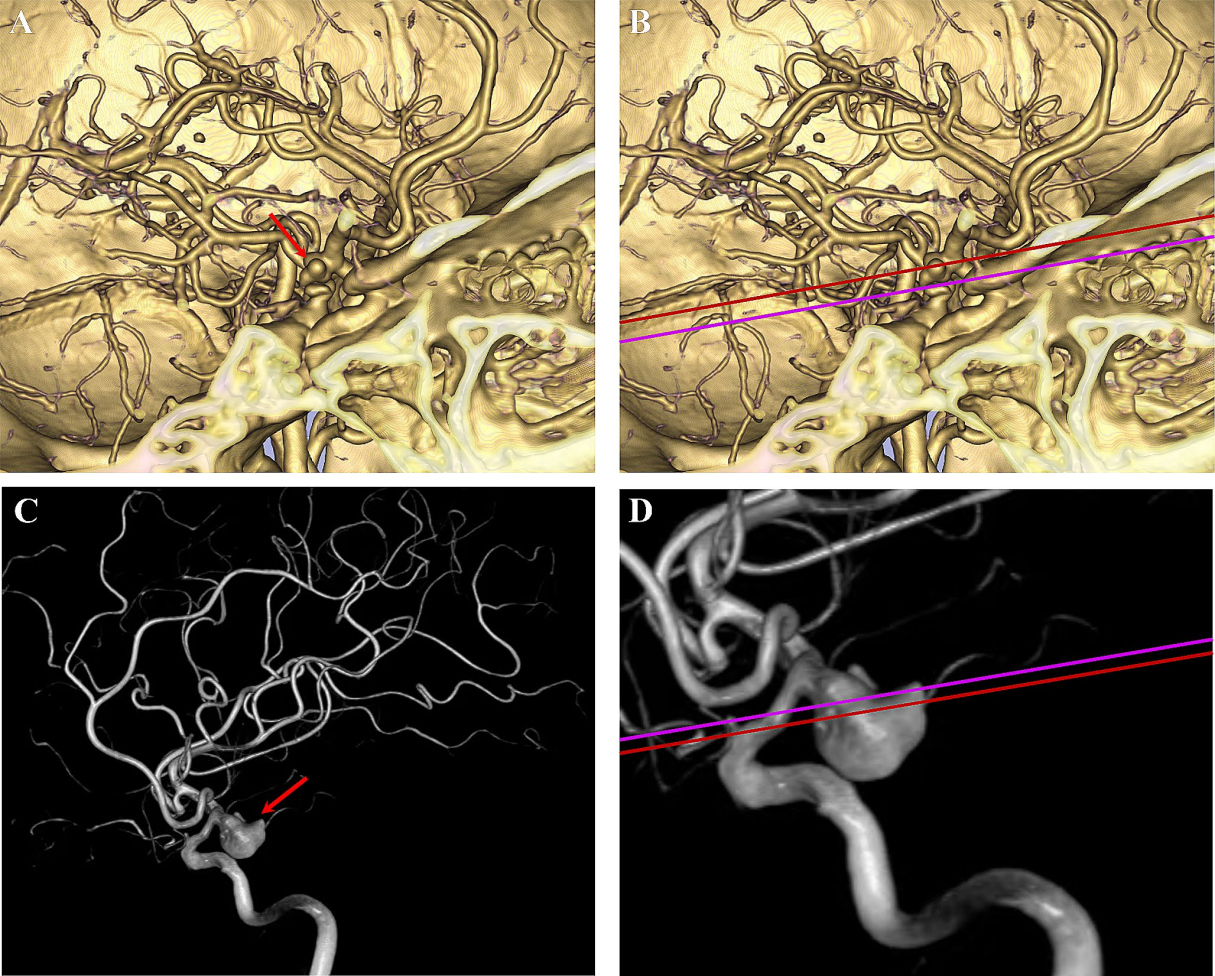
Diagram of AN. (**A**) The CTA image of a regular PCoA aneurysm (red arrow). (**B**) The level of SF is marked in pink, and the level of the aneurysmal neck is marked in red. The relative perpendicular distance between these two levels is AN. (**C**) The DSA image of another regular PcoA aneurysm (red arrow). (**D**) AN is the distance between the level of SF (pink line) and the aneurysmal neck (red line)

To calculate the distance-relevant parameters, we first defined the standard surface (SF). The SF is an imaginary plane that crosses the bilateral ACP tips and is parallel to the planum sphenoidale (Fig. 1). After determining the SF, distance-relevant factors were measured in millimeters as follows:

AN: The perpendicular distance between the proximal aneurysmal neck and the SF, where cases with an aneurysmal neck above the SF were assigned positive values, and those with an aneurysmal neck below the SF were assigned negative values (Fig. 2).

**Fig. 3:**
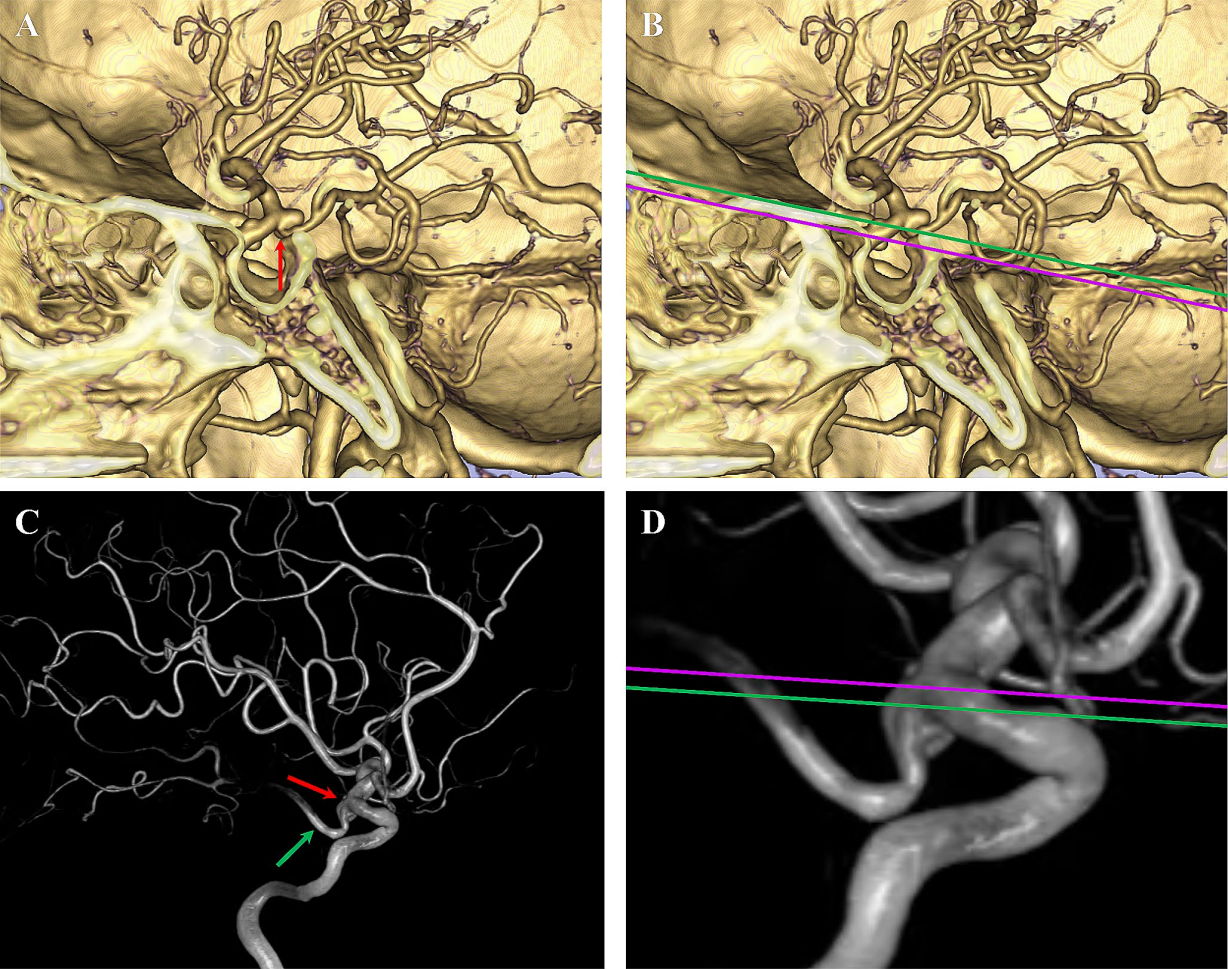
Diagram of AP. (**A**) The origin of the PCoA is clearly shown on CTA (red arrow). (**B**) The green line indicates the level of the PCoA origin. (**C**) Another PCoA aneurysm (red arrow) and the PCoA (green arrow) on DSA. (**D**) The AP is measured according to the level of SF (pink line) and the PCoA origin (green line)

AP: The perpendicular distance between the origin of the PCoA and the SF, where cases with the PCoA origin above the SF were assigned positive values, and those with the PCoA origin below the SF were assigned negative values (Fig. 3).

### Angle-relevant parameters (A to E)

**Fig. 4:**
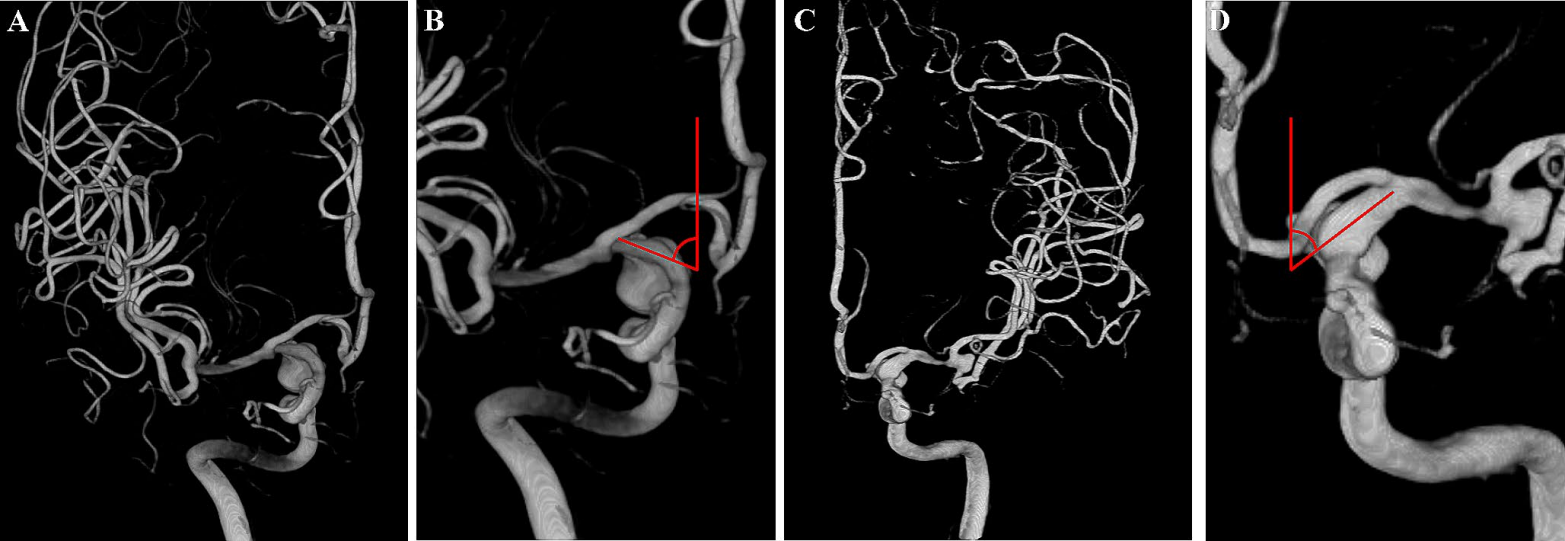
Diagram of angle A. Angle A was measured between a line perpendicular to the cranial base and the axis of the communicating segment of the ICA in the Towne anteroposterior view. **A**, **B**. A right-side PCoA aneurysm. **C**, **D**. A left-side PCoA aneurysm

Angle A was measured between a line perpendicular to the cranial base and the axis of the communicating segment of the ICA in the Towne anteroposterior view (Fig. 4).

**Fig. 5:**
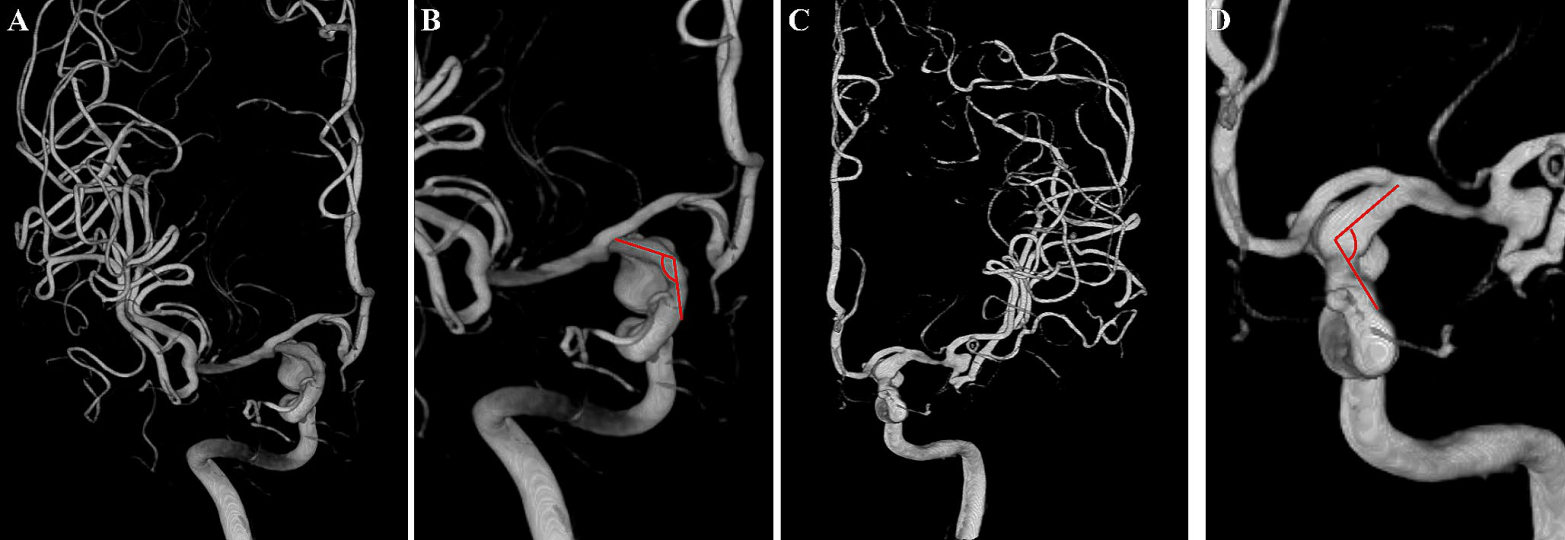
Diagram of angle B. Angle B was measured between the axes of the communicating and ophthalmic segments of the ICA in the Towne anteroposterior view. **A**, **B**. A right-side PCoA aneurysm. **C**, **D**. A left-side PCoA aneurysm. The ophthalmic arteries are clearly visible on the DSA images. However, the origins of the PCoAs are blocked by ICAs and aneurysms, leading to difficulties in precisely delineating the ophthalmic and communicating segments of the ICA

Angle B was measured between the axes of the communicating and ophthalmic segments of the ICA in the Towne anteroposterior view (Fig. 5).

**Fig. 6:**
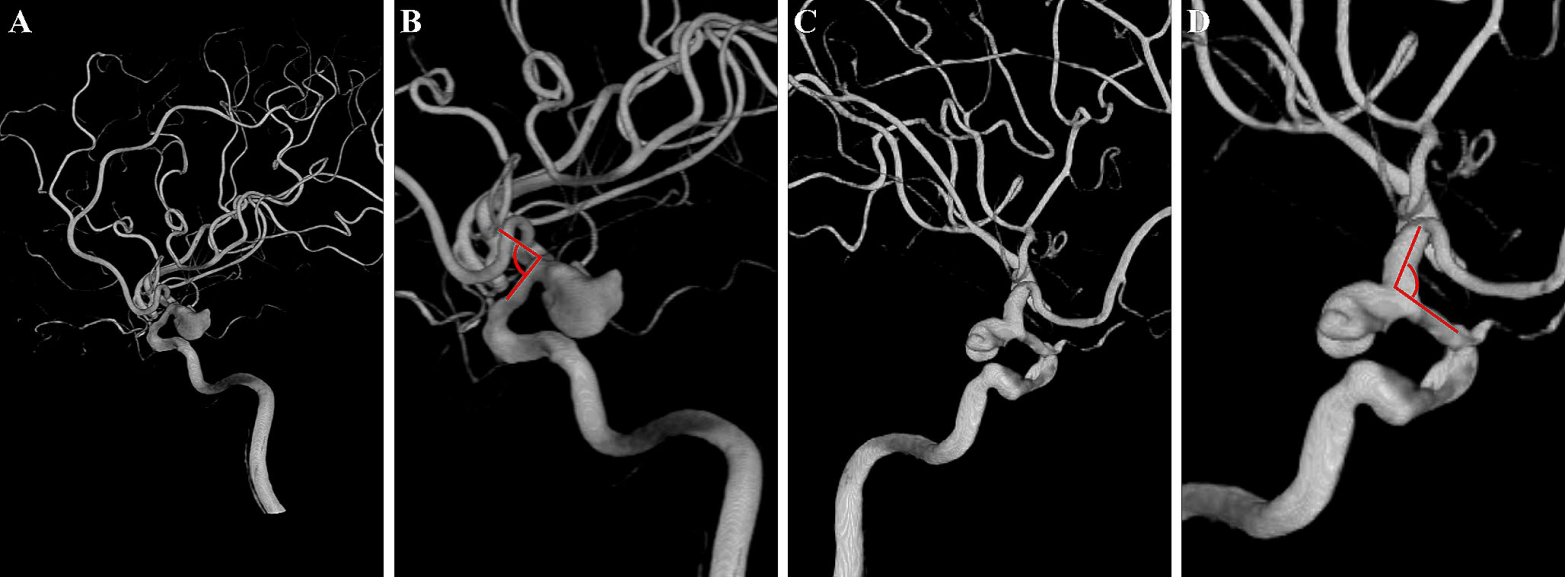
Diagram of angle C. Angle C was measured between the axes of the communicating and ophthalmic segments of the ICA in the Towne lateral view. The origins of the PCoAs are difficult to identify in the lateral view owing to the coverage of the aneurysms. **A**, **B**. A right-side PCoA aneurysm. **C**, **D**. A left-side PCoA aneurysm

Angle C was measured between the axes of the communicating and ophthalmic segments of the ICA in the Towne lateral view (Fig. 6).

The above methods for calculating angles A–C were reported by Niibo et al. in 2021 to predict the availability of proximal control of PCoA aneurysms [7], and we have used their definitions in our study. However, in practice, we found several limitations in accurately obtaining these angles based on DSA images. First, the origins of the PCoAs were sometimes entirely blocked by aneurysms, making precisely identify the ophthalmic and communicating segments of the ICA impossible. Second, the common anatomical variations of PCoAs, including the hypoplastic or aplastic type, also contributed to difficulties in accurately delineating PCoAs. Because the reported methods were relatively inaccurate and the measurement data were unstable, we proposed a modified method (angles D–E) to replace angles A–C.

**Fig. 7:**
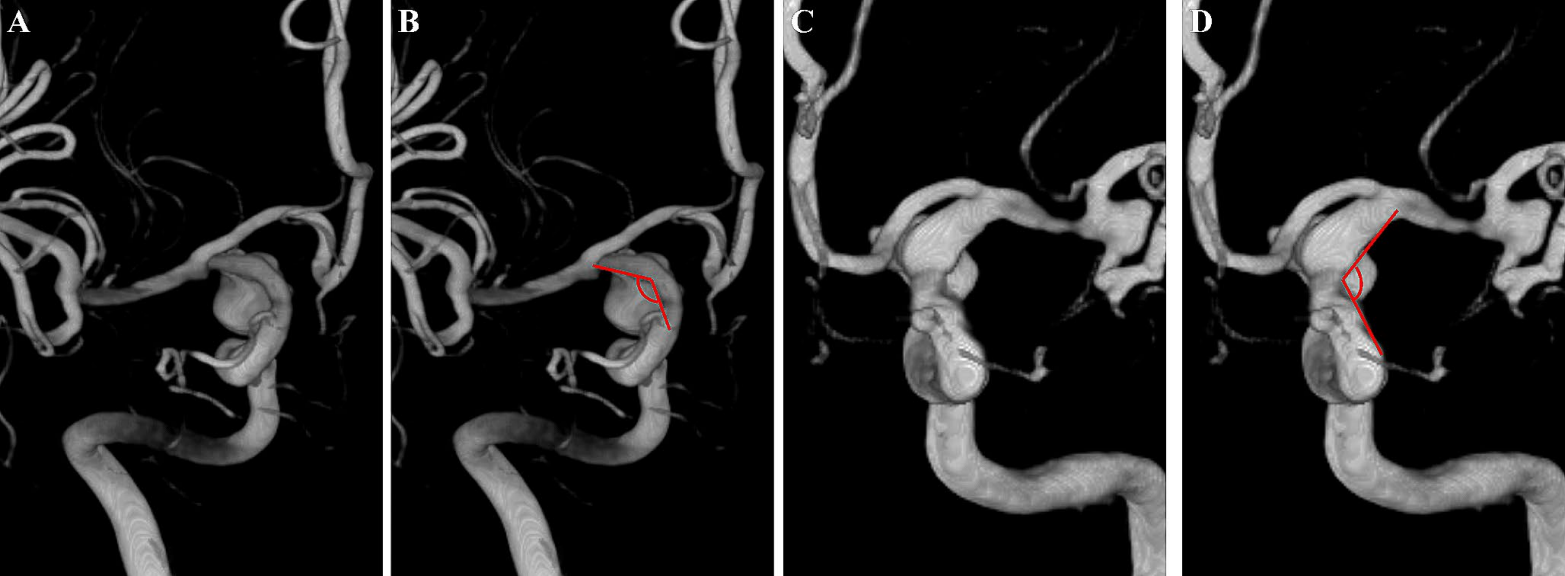
Diagram of angle D. Angle D represents the largest bending site during the course of the ophthalmic communicating segments of the ICA in the Towne anteroposterior view. Owing to the confusion regarding the accurate identification of the origin of the PCoA, we propose a modified method by selecting the most curved site along the lateral edge of the ICA. This simplified method is easier to follow and has improved accuracy. **A**, **B**. A right-side PCoA aneurysm. **C**, **D**. A left-side PCoA aneurysm

Angle D represented the largest bending site in the ophthalmic-communicating segments of the ICA in the Towne anteroposterior view (Fig. 7).

**Fig. 8:**
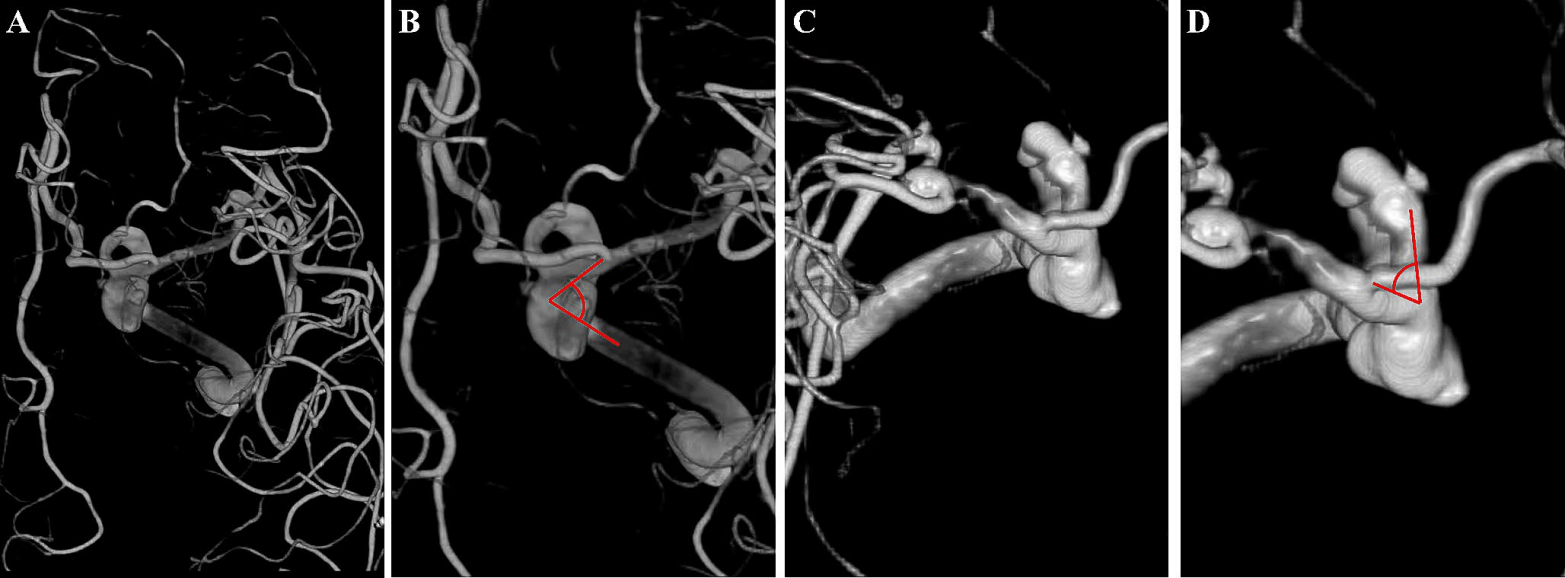
Diagram of angle E. Angle E represents the largest bending site in the course of the ophthalmic-communicating segments of the ICA from the top-down view. **A**, **B**. A right-side PCoA aneurysm. **C**, **D**. A left-side PCoA aneurysm

Angle E represented the largest bending site in the ophthalmic-communicating segments of the ICA in the top-down view (Fig. 8).

## Mathematical analysis

To integrate distance- and angle-relevant factors that reveal aneurysmal characteristics from different perspectives, we designed a prediction model using mathematical statistics. SPSS (version 27.0), and MATLAB (version R2022a) were used for the statistical analysis and construction of the mathematical prediction model. The clinical data between the “low-lying” and regular groups were compared using the Student’s t-test. Data are expressed as mean ± standard deviation (SD). Statistical significance was set at p-values < 0.05.

Multivariate logistic regression analysis was used to analyze the influence of different parameters. Furthermore, a prediction model of “low-lying” PCoA aneurysms was mathematically established by calculating the best weight-coefficient of each parameter. The receiver operating characteristic (ROC) curve was plotted, and the area under the curve (AUC) was analyzed to show the accuracy of the model. AUC values ranged from 0 to 1. Results were considered significant when the AUC was > 0.5. A model with an AUC of 1 indicated the best predictive ability.

### Microsurgical procedure

A pterional approach was used for each case. The Sylvian fissure was fully separated to expose the oculomotor nerve, ICA, PCoA, ACP, and APF. “Low-lying” PCoA aneurysms were intraoperatively identified using the following criteria: (1) aneurysms that were totally or partially covered by the ACP and APF; (2) the proximal portion of the ICA and aneurysmal neck were obscured by the ACP and APF; and (3) exposure of the aneurysmal neck and proximal control of the ICA were unfeasible without APF fenestration or anterior clinoidectomy. After identifying “low-lying” PCoA aneurysms, a 2–3 mm incision along the medial APF was performed and subsequently enlarged by bipolar coagulation to create a wedge-shaped fenestration corridor. A 1–3 mm anterior clinoidectomy was performed by high-speed drilling, when necessary, to fully visualize the aneurysmal neck, proximal portion of the ICA, and origin of the PCoA. Bone wax was applied after the anterior clinoidectomy to prevent bleeding and cerebrospinal fluid leakage.

The anatomical adhesions between the aneurysm, aneurysmal neck, APF, and penetrating vessels were carefully dissociated with a nerve dissector and micro-scissors. The proximal neck and APF were separated to determine the direction of the PCoA. The distal neck was separated, with a particular focus on preserving the adjacent PCoA-penetrating vessels and anterior choroidal artery. The oculomotor nerves were always located inferior to aneurysms. After the surrounding anatomical structures were clearly identified, temporary clipping of the proximal ICA was required to reduce the blood flow pressure within the aneurysm. Compatible clips were introduced into the aneurysmal neck, parallel to the ICA as much as possible, to avoid ICA stenosis. For most narrow-necked aneurysms, we selected standard aneurysm clips that were beneficial for exploring the integrity of the PCoA, perforator vessels, and oculomotor nerves after clipping. Standard or fenestrated clips were used to treat wide-necked aneurysms. When fenestrated clips were used, the PCoA and its perforating vessels required careful exploration to avoid mis-clipping. Severe adhesions between the oculomotor nerve and aneurysm, which should not be forcibly separated, were detected in some cases. Under these circumstances, the aneurysms were clipped and dissected for further separation from the oculomotor nerve. Intraoperative fluorescein angiography was used to confirm the absence of any residual aneurysmal neck and undisturbed blood flow in the ICA. Postoperative DSA was performed 2–4 days after the surgery to determine the clipping efficacy. After discharge, all patients were followed up at the outpatient center with CT angiography (CTA) examination.

## Results

This study included 89 patients with PCoA aneurysms, among whom 20 were classified into the “low-lying” group, and 69 were classified into the regular group.

### Clinical information

No significant differences were found in age, sex and GCS scores between the “low-lying” and regular groups. The average values of the distance parameters (AP and AN) and angle parameters (A–E) are summarized in Table 1. There were significant differences in AP, AN, angle D, and angle E between the two groups. These data were further analyzed to establish the predict model.

**Table 1:** Clinical data obtained from DSA results

Total:89 cases	Low-lying (*n* = 20)	Regular (*n* = 69)	*P*-value
Age, years	58.90 ± 8.15	61.41 ± 7.73	0.210
Male/Female	5:15	21:48	0.638
GCS	7.8 ± 2.36	8.2 ± 2.79	0.063
AP	0.11 ± 1.27	2.48 ± 1.68	<0.001
AN	-0.91 ± 0.76	1.31 ± 1.40	<0.001
Angle A	54.61 ± 15.38	47.33 ± 17.29	0.093
Angle B	110.75 ± 26.54	113.47 ± 22.90	0.559
Angle C	126.42 ± 18.39	132.77 ± 22.60	0.253
Angle D	90.12 ± 18.32	120.83 ± 21.12	<0.001
Angle E	97.47 ± 22.31	111.10 ± 17.79	0.006

### Prediction model

Seven parameters that reflected different aspects of aneurysmal characteristics were incorporated into the logistic regression analysis. The results showed that the AN and angle D were significantly correlated with the presence of “low-lying” aneurysms (Table 2). The probability function was obtained using the SPSS software. The accuracy of the probability function was subsequently tested and analyzed using MATLAB. The probability formula was as follows:

**Table 2:** Multivariate logistic regression analysis of seven parameters in the prediction model

	Coefficient	*P*-value
AP	0.332	0.679
AN	-2.789	0.031
Angle A	-0.021	0.577
Angle B	0.005	0.819
Angle C	-0.066	0.065
Angle D	-0.114	0.003
Angle E	0.052	0.162

$$P\, = \,{{{e^{0.332AP - 2.789AN - 0.021A + 0.005B - 0.066C - 0.114D + 0.052E + 11.374}}} \over \matrix{1 + \hfill \cr {e^{0.332AP - 2.789AN - 0.021A + 0.005B - 0.066C - 0.114D + 0.052E + 11.374}} \hfill \cr} }$$

**Fig. 9:**
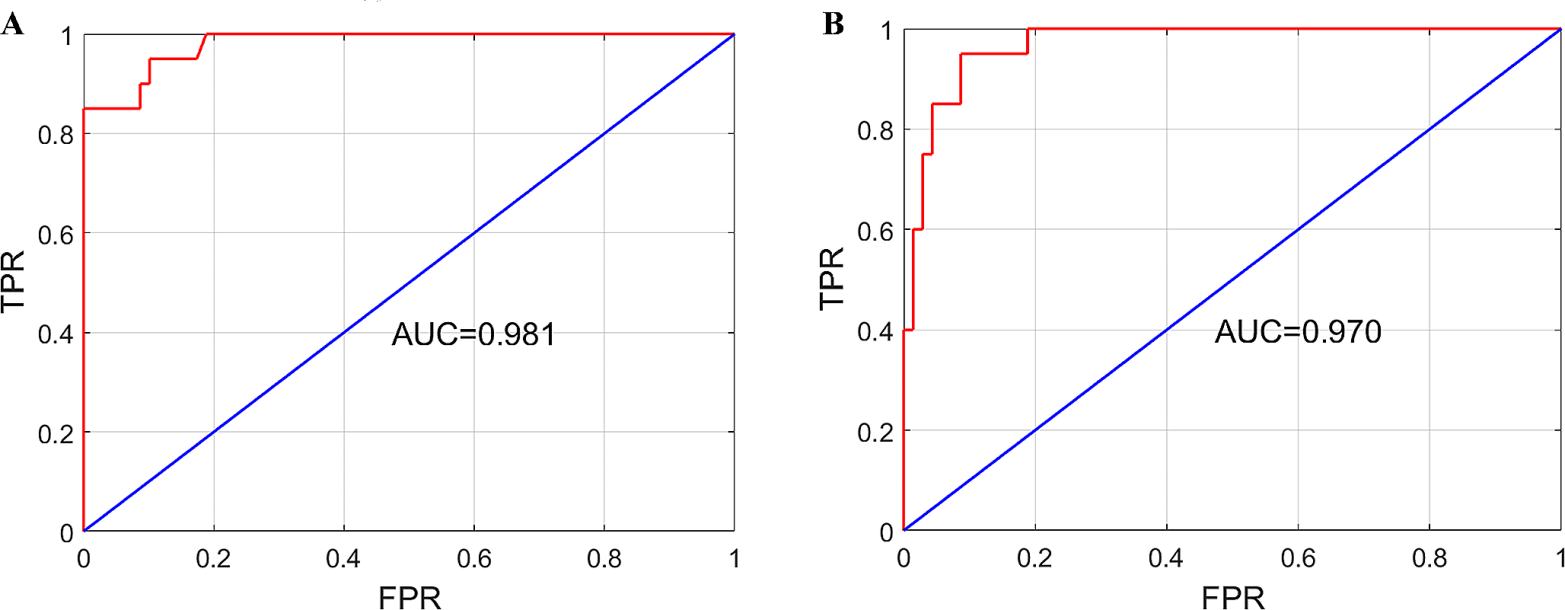
Mathematical statistics of the prediction model. (**A**) The ROC curve of the prediction model with seven parameters. (**B**) The ROC curve of the simplified prediction model with two parameters

This formula represents the probability of a “low-lying” aneurysm, where “e” is the Napierian logarithm. The accuracy, sensitivity (TPR), and specificity (FPR) of the prediction model were verified using a confusion matrix. The AUC of the ROC curve was 0.981, and the prediction accuracy was 94.4%, indicating the superior predictive ability of this model (Fig. 9A).

For the convenience of actual practice, we proposed a modified model that was more concise. In the modified model, we selected only two predictors (AN and angle D) with proven significance (Table 2). We reintegrated these two factors into the logistic regression analysis, and the significant differences were consistent with previous results (Table 3). Following the aforementioned method, we obtained the following simplified probability equation:

**Table 3:** Multivariate logistic regression analysis of two parameters in the simplified prediction model

	Coefficient	*P*-value
AN	-1.862	< 0.001
Angle D	-0.105	0.002

$$\:P=\frac{{e}^{-1.862AN-0.105D+9.910}}{1+{e}^{-1.862AN-0.105D+9.910}}$$

The AUC was 0.970, and the prediction accuracy was 93.2%, indicating that the modified model also possessed reliable predictive ability (Fig. 9B). Because the Napierian logarithm is equal to approximately 2.718, the formula was further simplified to

$$\:P=\frac{{2.718}^{-1.862AN-0.105D+9.910}}{1+{2.718}^{-1.862AN-0.105D+9.910}}$$

## Discussion

### APF and PcoA

The APF is an extension of the anteromedial tentorial edge, to which the ophthalmic and communicating segments of the ICA can be identified medially. Along the course of the APF, the oculomotor nerve is located medially at the oculomotor triangle, which is also defined as the posterior roof of the cavernous sinus [8]. Notably, venous outflow from the cavernous sinus has been detected within the APF, which explains the venous bleeding during APF incisions [9]. The internal sinus also indicates the thickness of the APF.

The PcoA originates from the dorsal aspect of the ICA and follows the posteromedial direction, superior to the oculomotor nerve, to join the PCA. The entire course of the PcoA is located inside the anterior incisural space, from the anterolateral carotid cistern to the posteromedial interpeduncular cistern, with a mean length of 12.5 mm [10, 11]. Considering the structure of the oculomotor triangle and the adjacent relationship between the oculomotor nerve and the PcoA, it is easier to notice that the PcoA runs above and medial to the APF and the free margin of the tentorium cerebelli. In general, the origin of the PcoA cannot be occluded by the APF. However, under particular circumstances, including anatomical variations or arteriosclerosis, the elasticity and curvature of the intracranial ICA are changed. The lateral edge of the communicating segment of the ICA is in close proximity to the APF. The origins of the PcoA and proximal aneurysmal neck are more likely to be obscured by APF in these cases, resulting in the appearance of “low-lying” PcoA aneurysms.

### “Low-lying” PcoA aneurysms

ICA-PcoA aneurysms are the most common type of intracranial aneurysm. Most PcoA aneurysms originate distal to the origin of the PcoA; therefore, tracing the posterolateral wall of the proximal ICA is an important step in identifying the origin of the PcoA and aneurysmal neck. “Low-lying” PcoA aneurysms are also defined as posterolaterally projecting PcoA aneurysms owing to their unique positions and directions [5, 12]. Despite symptoms related to subarachnoid hemorrhage, “low-lying” aneurysms are more likely to show preoperative oculomotor paralysis as a result of infratentorial projecting [13]. The proximal necks of “low-lying” PcoA aneurysms are under the level of the ACP and are entirely or partially occluded by the APF, which leads to increased difficulties in tracing the proximal ICA and origin of the PcoA. Once the “low-lying” PcoA aneurysms are confirmed, APF coagulation is necessary to release an additional 2–3 mm of space to uncover the hidden aneurysmal neck. Anterior clinoidectomy should also be considered to expose the extracavernous portion of the ICA if extra space is required for proximal control and successful clipping. Accurately identifying the PcoA and surrounding perforators is necessary to prevent postoperative infarctions. In addition, achieving proximal and distal control before attempting clipping is important. Considerable attention is required to explore the aneurysmal sac and correct the clip position, ensure that no parent stem or surrounding vessels are influenced, and evaluate the necessity of adding extra clips for successful clipping.

### Prediction model of “low-lying” PcoA aneurysms

Preoperatively identifying “low-lying” PcoA aneurysms is important to estimate surgical complexities and avoid complications from clipping. Some clinical studies have reported their methods of predicting “low-lying” PcoA aneurysms according to the relationship with the APF, ACP, and adjacent soft tissues [7, 14]. Niibo et, al. [7] proposed a sophisticated system for predicting anatomical variations in PcoA aneurysms. Distance factors considering the aneurysm features and angle factors reflecting the tortuosity of the ICA were obtained from preoperative CTA images and simultaneously incorporated into the criterion. The short distance between the proximal aneurysmal neck and the ACP tip and calcification of the ophthalmic segment of the ICA were the strongest predictors for assessing the difficulties of proximal control in “low-lying” PcoA aneurysms. However, angle-related data have failed to provide valuable details for the operation [7]. Another study introduced a simple method to mark the APF based on CTA results. The anatomical line between the base of the ACP and the superior edge of the arcuate eminence in the lateral view corresponded to the actual direction of the APF. Therefore, “low-lying” PcoA aneurysms can be identified preoperatively in terms of the relative localization with the APF [15].

These studies greatly contributed to predicting “low-lying” PcoA aneurysms. However, these methods had some limitations. For example, identifying the arcuate eminence on DSA examinations is difficult, which makes precisely determining the location of the APF difficult. In reality, the APF thickness further limits the prediction accuracy of a single connecting line. In addition, during the application of Niibo’s methods in practice, we found that the angle-relevant results were somewhat more subjective than the distance-relevant data. Impeccable identification of the ophthalmic and communicating segments of the ICA was challenging, as the origins of the PcoAs were sometimes blocked by aneurysms. A slight bias in determining the axis of the ICA segments would generate significant deviations in angle-relevant data. We suspect that the unstable measurement of the angles might have concealed their actual contributions to the prediction. As DSA is the gold standard for aneurysm diagnosis, we proposed a modified system using DSA results that further promoted the accuracy and operability for neurosurgeons to predict “low-lying” PcoA aneurysms.

**Fig. 10:**
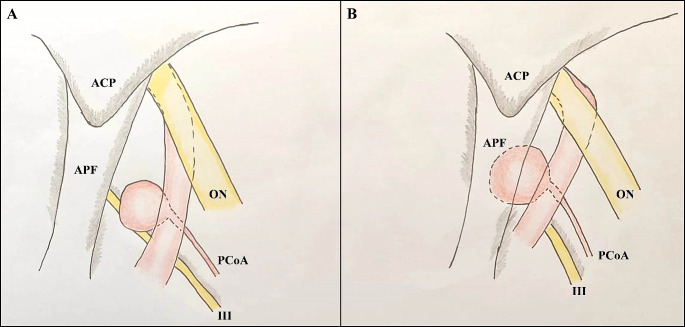
The anatomic relationship between the APF and communicating segment of ICA. Cartoon diagram showing the relationship between different PCoA aneurysms and adjacent structures from the axial plane at the level of the ACP. The relative tortuosity of the ophthalmic and communicating segments of ICA is related to the presence of “low-lying” PCoA aneurysm to some extent. (**A**) The regular PCoA aneurysm with less tortuosity of ICA. (**B**) The “low-lying” PCoA aneurysm with relatively large tortuosity of ICA. The aneurysmal neck is under the level of ACP and a large proportion of the aneurysm is covered by the APF

We chose the planum sphenoidale and bilateral ACPs to standardize the SF, AP and AN were measured accordingly. As most APFs are located under the SF level, negative AN values, which indicate lower aneurysmal neck positions, cannot directly indicate a “low-lying” type. APF thickness also contributed to larger errors when AN was selected as a single predictor. To increase the accuracy of the prediction data, we added angle-relevant factors to depict the ICA tortuosity in our model. Statistical analysis showed that the angle D greatly influenced the prediction accuracy, together with AN. When the aneurysmal necks were located under the SF (i.e., negative AN values), the smaller angle D indicated the greater likelihood of “low-lying” aneurysms. The anatomical meaning of the mathematical results can be clarified by understanding the relationship between the APF and the communicating segment of the ICA. A smaller angle D in the Towne anteroposterior view reveals a relatively larger degree of binding between the ophthalmic and communicating segments of the ICA. In the Towne anteroposterior view, the axis of the communicating segments of the ICAs with a smaller angle D was more likely to be directed toward the tentorial edge and ACP (Fig. 10). This anatomical relationship partly explains why the origins of PcoAs or aneurysmal necks are more likely to be covered by the APF and ACP in cases with a negative AN value and smaller angle D.

**Fig. 11:**
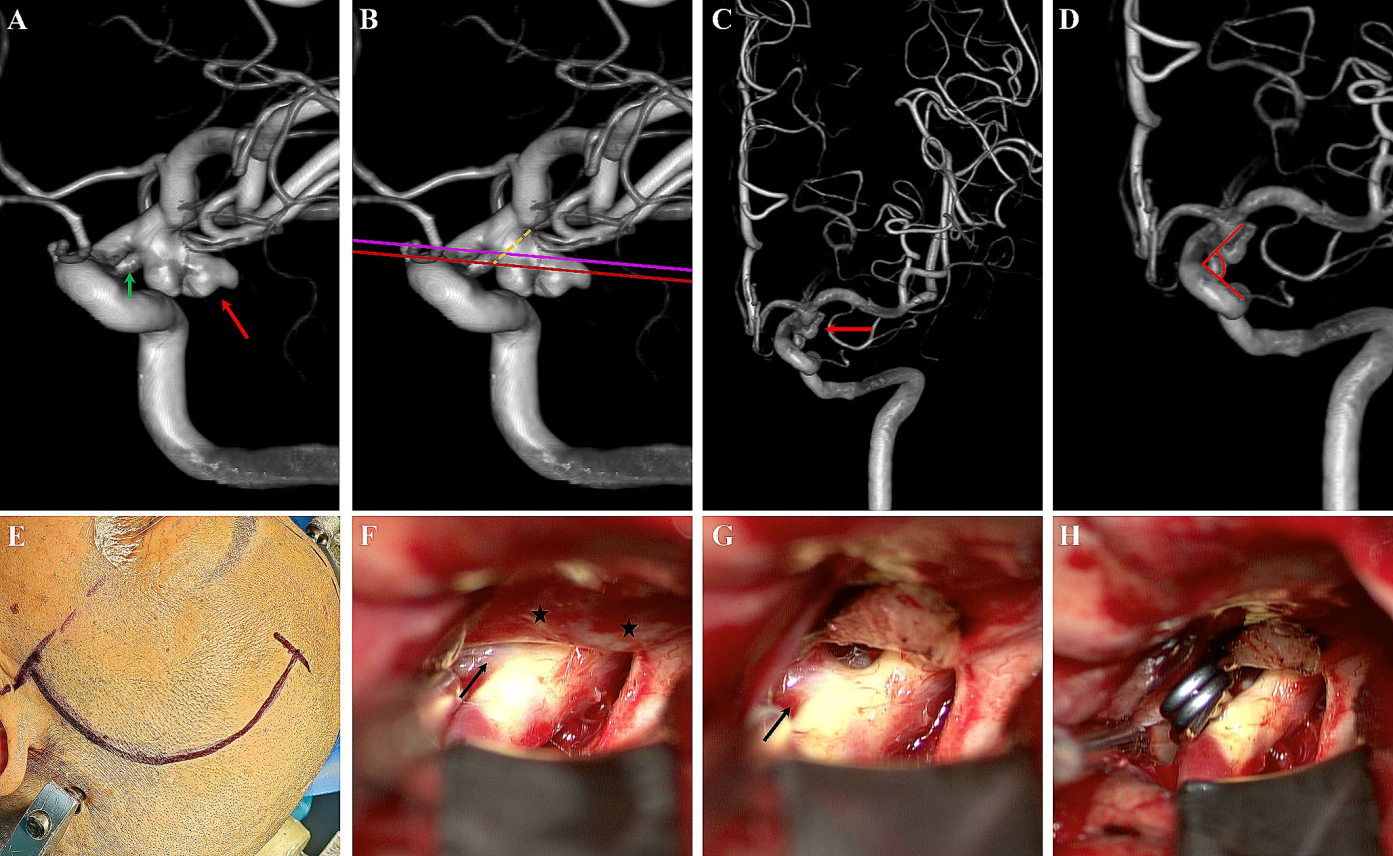
Applying the simplified model to predict “low-lying” PCoA aneurysms. (**A**) DSA examination shows a PCoA aneurysm on the left (red arrow: aneurysm; green arrow: PCoA). (**B**) The proximal aneurysmal neck and SF are marked in red and pink, respectively. The yellow dotted line indicates the direction of clipping in the operation. AN (-1.44 mm) was calculated accordingly. **C**, **D**. Angle D (87.6°) was measured along the largest bending site of the ICA. The predictive probability was calculated by integrating AN and angle D into the prediction formula. The final predictive probability was 0.96749, indicating a great probability of a “low-lying” aneurysm (red arrow: aneurysm). **E**. The standard pterion approach was performed. **F**. The optic nerve and ophthalmic segment of the ICA were exposed after separating the Sylvian fissure. The “low-lying” PCoA aneurysm was confirmed, and a large proportion of the aneurysm was covered by the APF (arrow: aneurysm, star: APF). **G**. The aneurysmal neck and proximal ICA were exposed after APF fenestration. **H**. Permanent clipping was performed after clearly identifying the surrounding anatomic structures

Our study presented a modified model to predict the existence of “low-lying” PcoA aneurysms. The simplified model is convenient to follow because it includes only two factors. AN and angle D data were obtained from the 3D-DSA images without additional calculations. According to our practice, all measurement and calculation procedures could be completed in 3 min using the simplified prediction formula (Fig. 11). Neurosurgeons and radiologists might benefit from this method that allows them to directly recognize “low-lying” PcoA aneurysms and make appropriate surgical decisions accordingly. The selection of microsurgical clipping or endovascular embolization should be adequately evaluated based on the shape, size, and patient condition in cases of “low-lying” PcoA aneurysms.

### Limitations

This study had some limitations. First, in today’s context, CTA is more available for cerebrovascular surgeons for microsurgical planning, especially in emergency situations. However, the model was established based on DSA data, and there were relatively large errors when compared the relevant CTA parameters with the DSA data due to the blockade of osseous structures and the blurred CTA images in some cases. In this regard, this model cannot be directly replicated in CTA examinations. A novel model corrected by CTA data is required in future study. Second, although we attempted to introduce an improved method to reduce subjective bias in measuring angle-relevant parameters, the angle factor data, especially angle D, still showed some subjective differences when measured by different surgeons. Moreover, the statistical power of the prediction model was limited because of the sample size.

## Conclusion

This novel model allows the preoperative prediction of “low-lying” PcoA aneurysms based on DSA results that is strongly consistent with surgical findings. This prediction model enables neurosurgeons to comprehensively identify the presence of “low-lying” PcoA aneurysms by simply reviewing preoperative DSA images. Furthermore, this model assists us in further understanding the distinct anatomical relationships between “low-lying” PcoA aneurysms and surrounding structures.

## Data Availability

No datasets were generated or analysed during the current study.

## References

[1] Rosato R, Comptdaer G, Mulligan R et al (2020) Increased focal internal carotid artery angulation in patients with posterior communicating artery aneurysms. J Neurointerv Surg 12:1142–1147 32447300 10.1136/neurintsurg-2020-015883

[2] Spetzler RF, Zabramski JM, McDougall CG et al (2018) Analysis of saccular aneurysms in the Barrow ruptured Aneurysm Trial. J Neurosurg 128:120–125 28298031 10.3171/2016.9.JNS161301

[3] Shi L, Yu J, Zhao Y, Xu KYu J (2018) Clipping treatment of posterior communicating artery aneurysms associated with arteriosclerosis and calcification: a single center study of 136 cases. Exp Ther Med 15:1647–1653 29434749 10.3892/etm.2017.5525 PMC5774401

[4] Kim JH, Kim JM, Cheong JH, Bak KHKim CH (2009) Simple anterior petroclinoid Fold resection in the treatment of low-lying internal carotid-posterior communicating artery aneurysms. Surg Neurol 72:142–145 18789509 10.1016/j.surneu.2008.03.045

[5] Ji S, Shi X, Chu X et al (2017) Surgically clipping a Posterolaterally projecting posterior communicating artery aneurysm with Anterior Petroclinoid fold Fenestration. J Craniofac Surg 28:e47–e49 27893552 10.1097/SCS.0000000000003197

[6] Byoun HS, Choi KS, Na MK, Kwon SMNam YS (2023) The usefulness of Extradural Anterior Clinoidectomy for Lower-lying posterior communicating artery aneurysms: a cadaveric study. J Korean Neurosurg Soc. 10.3340/jkns.2023.0184 38061762 10.3340/jkns.2023.0184 PMC11220413

[7] Niibo T, Takizawa K, Sakurai J et al (2020) Prediction of the difficulty of proximal vascular control using 3D-CTA for the surgical clipping of internal carotid artery-posterior communicating artery aneurysms. J Neurosurg 134:1165–1172 32276244 10.3171/2020.1.JNS192728

[8] Umansky F, Valarezo AElidan J (1994) The superior wall of the cavernous sinus: a microanatomical study. J Neurosurg 81:914–920 7965122 10.3171/jns.1994.81.6.0914

[9] Spencer PS, Cardona JJ, Reina F et al (2023) A newly discovered dural venous sinus of the Skull Base: the Anterior Petroclinoid Sinus. World Neurosurg 172:e581–e584 36716855 10.1016/j.wneu.2023.01.087

[10] Pescatori L, Taurone S, Ciccarelli A et al (2023) Petroclival Clinoidal Folds and Arachnoidal membranes of the Anteromedial Incisural Space: clinical anatomy for neuro critical care. Diagnostics (Basel). ; 13 10.3390/diagnostics13203203 PMC10605941 37892024

[11] Romero Adel C, da Silva CEde Aguiar PH (2011) The distance between the posterior communicating arteries and their relation to the endoscopic third ventriculostomy in adults: an anatomic study. Surg Neurol Int 2:91 21748043 10.4103/2152-7806.82373 PMC3130471

[12] Thiarawat P, Jahromi BR, Kozyrev DA et al (2017) Microneurosurgical Management of posterior communicating artery aneurysm: a contemporary series from Helsinki. World Neurosurg 101:379–388 28213191 10.1016/j.wneu.2017.02.033

[13] Gonzalez-Darder JM, Quilis-Quesada V, Talamantes-Escriba F, Botella-Macia LVerdu-Lopez F (2012) Microsurgical relations between Internal Carotid artery-posterior communicating artery (ICA-PComA) segment aneurysms and Skull Base: an Anatomoclinical Study. J Neurol Surg B Skull Base 73:337–341 24083126 10.1055/s-0032-1322795 PMC3578643

[14] Yoon S, Kim MJ, Han HJ et al (2023) Added predictive values of Proton Density Magnetic Resonance Imaging on posterior communicating artery aneurysms and surrounding soft tissues with simple classification. J Korean Neurosurg Soc 66:418–425 36588389 10.3340/jkns.2022.0259 PMC10323277

[15] Akamatsu Y, Kashimura H, Fujiwara S, Kubo YOgasawara K (2019) Simple assessment of the localization of posterior communicating artery aneurysms to the anterior petroclinoid ligament. J Clin Neurosci 66:38–40 31153753 10.1016/j.jocn.2019.05.030

